# An active learning-based approach for screening scholarly articles about the origins of SARS-CoV-2

**DOI:** 10.1371/journal.pone.0273725

**Published:** 2022-09-16

**Authors:** Xin An, Mengmeng Zhang, Shuo Xu

**Affiliations:** 1 School of Economics & Management, Beijing Forestry University, Beijing, P.R. China; 2 College of Economics and Management, Beijing University of Technology, Beijing, P.R. China; University of Engineering and Technology Taxila Pakistan, PAKISTAN

## Abstract

To build a full picture of previous studies on the origins of SARS-CoV-2 (severe acute respiratory syndrome coronavirus 2), this paper exploits an active learning-based approach to screen scholarly articles about the origins of SARS-CoV-2 from many scientific publications. In more detail, six seed articles were utilized to manually curate 170 relevant articles and 300 nonrelevant articles. Then, an active learning-based approach with three query strategies and three base classifiers is trained to screen the articles about the origins of SARS-CoV-2. Extensive experimental results show that our active learning-based approach outperforms traditional counterparts, and the uncertain sampling query strategy performs best among the three strategies. By manually checking the top 1,000 articles of each base classifier, we ultimately screened 715 unique scholarly articles to create a publicly available peer-reviewed literature corpus, *COVID-Origin*. This indicates that our approach for screening articles about the origins of SARS-CoV-2 is feasible.

## 1. Introduction

In December 2019, a novel coronavirus SARS-CoV-2 caused a serious outbreak of acute respiratory disease [1]. This has brought the epidemic into the field of vision of human beings again, and the outbreak is still ongoing in many countries and territories. To completely block the spread of the epidemic and to further prevent similar or more serious epidemics in the future, the most fundamental task is to find the origins of SARS-CoV-2 and clarify how it reaches the human population [2]. In human history, the sources of many viruses are very difficult to trace [3,4]. For the purpose of successfully tracing the origins of a virus, multiple steps are involved in this procedure: epidemiological investigation, genome analysis, intermediate and natural host identification, field sampling, homology analysis of the virus strain and so on. To resolve this complicated puzzle, it is necessary for scientists to build a full picture of previous studies on the origins of SARS-CoV-2 and remain up to date on the latest ones.

In the literature, there is an explosive growth in scientific publications on COVID-19 (Corona Virus Disease 2019) and SARS-CoV-2 [5–7]. Zuo et al. [8] observed that 128 unique datasets on SARS-CoV-2 and COVID-19, including LitCovid [9] and CORD-19 [10], have been developed and updated regularly for different tasks. However, to the best of our knowledge, there is no benchmark literature dataset publicly available for the origins of this virus. To bridge this gap and to promote global cooperation, this study is devoted to screening scholarly articles about the origins of SARS-CoV-2 from large numbers of scientific publications.

A naïve solution for screening articles is to carefully design a search strategy and then to retrieve the related publications from a comprehensive bibliographic database. Although this solution is very popular in practice, precision and recall are still the most concerning issues [11,12]. Take the strategy “TS = (SARS-CoV-2) AND TS = (origin)” in the Web of Science as an example. Several irrelevant articles, such as [13,14], appear in the result list since their titles or abstracts simultaneously contain the keywords *SARS-CoV-2* and *origin*. In addition, multiple relevant publications are missed, such as [15,16]. An alternative solution is to see document screening as a binary text classification problem. However, many text classification methods rely on the availability of a large labeled corpus. Due to an unprecedented volume of academic articles published related to this epidemic, it is not realistic to manually annotate enough samples for a text classification method with satisfactory performance. To meet this challenge, this study proposes an active learning-based approach for screening scholarly articles about the origins of SARS-CoV-2 with the following main contributions:

- An active learning-based approach is proposed to screen scientific publications about the origins of SARS-CoV-2.
- A curated peer-reviewed literature corpus (COVID-Origin), which can be freely accessed at https://github.com/pzczxs/COVID-Origin, was developed to track up-to-date peer-reviewed studies on the origins of SARS-CoV-2.
- Extensive experiments indicate that our approach, especially armed with multiple base classifiers, can efficiently screen scholarly articles about the origins of SARS-CoV-2.

The rest of the article is organized as follows. After briefly reviewing related work in Section 2, we describe the detailed process of data collection, annotation, and document representation in Section 3. Then, an active learning-based framework is developed in more detail in Section 4, and extensive experiments are conducted in Section 5. Section 6 concludes this contribution with the possible limitations of our study and future research.

## 2. Related work

### 2.1. Automatic document screening

As its name states, automatic document screening automatically finds all relevant documents pertinent to a given topic. Hence, this problem is also referred to as *the total recall problem* in the field of information retrieval [17]. More specifically, this problem can be formally described as follows. Given a set of candidate documents, of which only a small fraction is positive, each candidate can be checked to determine its label as positive or negative. The task is to check and label as few candidates as possible while achieving very high recall.

Since the work of Counsell [18], many approaches have been developed in the literature. It is well motivated in many applications, including systematic reviews in evidence-based medicine [19,20] and software engineering [21,22] and electronic discovery in legal proceedings [23]. In addition, several recent challenges, such as TREC [17,24] and CLEF eHealth task 2 [25–27], further promote the development of automatic document screening. To the best of our knowledge, two main research branches can be observed in the literature: information retrieval and machine learning.

In the area of information retrieval, the related investigations can be further divided into three groups: relevant feedback [20,28,29], query expansion [29–31] and ranking learning [32–34]. The former two methods emphasize transforming or improving the original query. The main difference is that relevant feedback is devoted to gathering information representing the user’s need and automatically creating a new query, and query expansion reformulates a given query with synonyms or semantically related terms to match additional documents. The ranking learning methods sort all documents so that the relevant documents are ranked before irrelevant ones as many as possible.

In fact, document screening can also be regarded as a binary classification problem (*relevant* versus *nonrelevant*). In theory, any supervised machine learning model for text classification can be utilized directly, such as naïve Bayes [35], support vector machines [36,37], random forests [37] and so on. However, due to the severe imbalance of relevant and nonrelevant instances, time-consuming annotation and heavy workload, the performance of many supervised models is not satisfactory. In recent years, considerable effort has been spent on screening documents with *active learning* strategies [38–40]. The main idea of this strategy is that a supervised model can perform better with fewer annotated instances if it is allowed to choose the instances from which it learns [41]. It has been shown that this active learning solution outperforms its counterparts in many real-world cases [17,24–27]. Therefore, we adopted an active learning algorithm to screen scholarly articles about the origins of SARS-CoV-2.

### 2.2. Active learning

In many real-world applications, large numbers of unannotated instances are easily available, but annotated instances are time-consuming and expensive to obtain. In such a scenario, a machine learning algorithm can actively query an oracle (e.g., a human annotator) for the label of a focal instance. This type of iterative supervised learning method is called *active learning* [41]. It is sometimes referred to as *optimal experimental design* or *query learning* in the statistics literature [42]. The overall goal is to construct a classifier as good as possible with fewer labeled instances than necessary [43].

**Fig 1:**
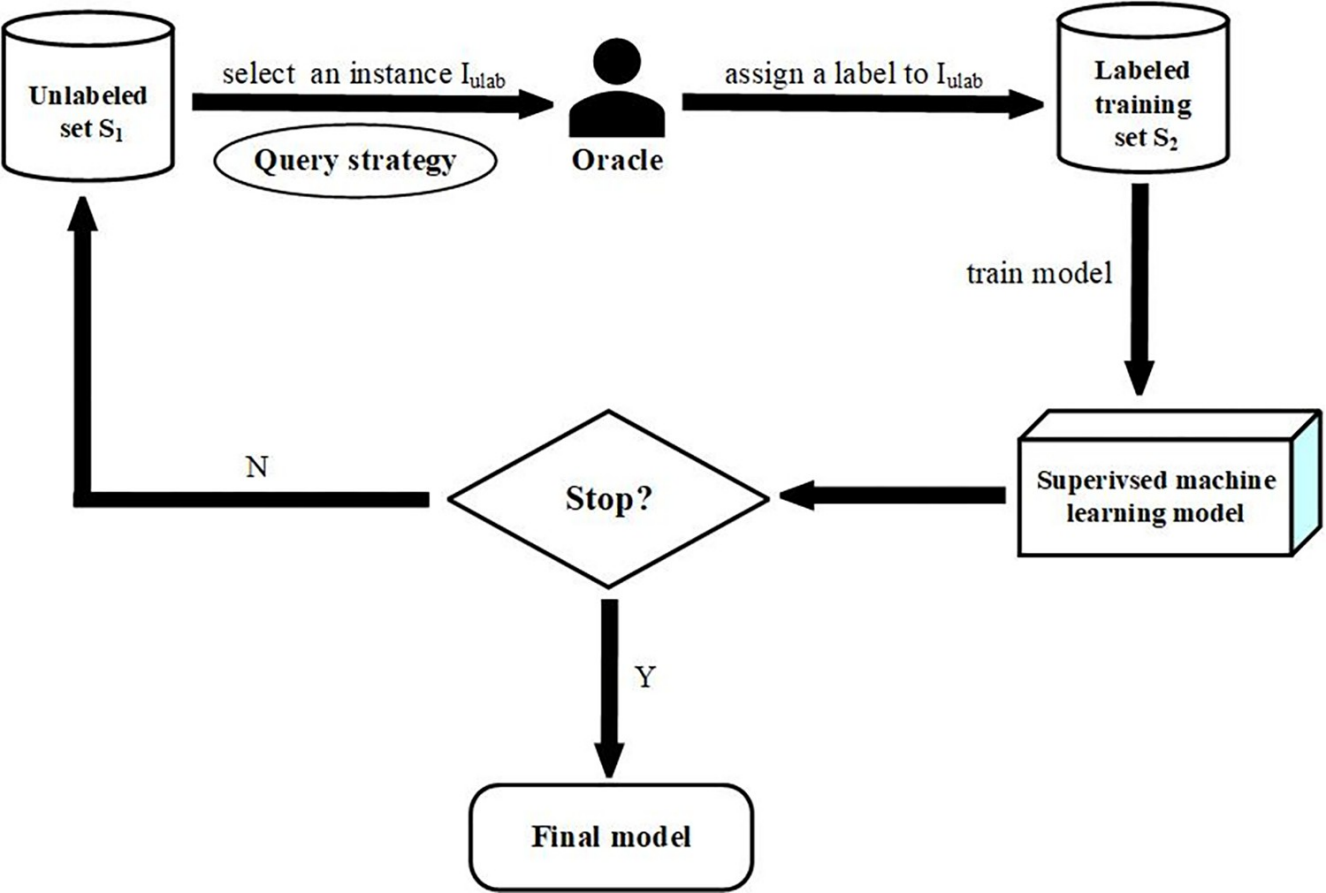
The procedure of an active learning approach.

Active learning mainly consists of five steps, as illustrated in Fig 1. Given an unlabeled set *S*_1_, these steps will be described briefly as follows.

**Step 1** A labeled training set is initialized to an empty set, i.e., *S*_2_←∅.
**Step 2** One *query strategy* is utilized to select the most valuable instance *I*_*ulab*_ from *S*_1_, and then a label is assigned by an oracle to this instance *I*_*ulab*_.
**Step 3** The instance *I*_*ulab*_ with its label is added to the training set, viz. *S*_2_←*S*_2_∪{*I*_*ulab*_} and removed from the unlabeled set, viz. *S*_1_←*S*_1_−{*I*_*ulab*_}.
**Step 4** A supervised machine learning model is retrained on the updated set *S*_2_.
**Step 5** Steps 2–4 are looped until a stopping criterion is met.

In Step 2, a variety of query strategies are put forward in the literature, such as uncertain sampling [44], expected error reduction [45], and query by committee [46,47]. The uncertain sampling query strategy, as its name implies, selects the instance on whose label the classifier is most uncertain. The expected error reduction strategy is devoted to annotating an instance so that the current classifier can achieve a lower generalization error. Different from the former two strategies, the query by committee strategy simultaneously considers multiple classifiers (viz. a committee of classifiers) and operates by querying the label of the instance on which these classifiers disagree the most. Santos et al. [48] comprehensively compared the pros and cons of these query strategies on a large number of datasets and suggested that uncertain sampling and expected error reduction strategies should be preferred in many real-world scenarios.

In Step 3, a supervised machine learning model is involved. Theoretically speaking, any supervised classification model can be used in this step. However, due to different performance, time and space complexities, the following models were deployed in previous studies: support vector machine (SVM) [49–51], random forest (RF) [52], naïve Bayes classifier [48,53], and logistic regression (LR) [48,53].

In Step 4, the most important thing is when to stop this iterative procedure. Cormack et al. [23] argued that enough annotated instances should be seen as a signal to stop learning. In fact, it is usually very difficult to determine how many annotated instances are sufficient in real-world applications. Therefore, many scholars have considered whether a focal model approaches stability in terms of performance as a stopping criterion [43,54]. The measures for performance include the F_1_ score, the area under the receiver operator characteristic (ROC) curve or the precision-recall (PR) curve.

## 3. Datasets

### 3.1. Data collection and annotation

#### Data collection

Due to the difficulty and complexity of the traceability of SARS-CoV-2, the available scientific publications are very scarce in the literature. Domingo [55] found only 1,675 results in the PubMed database with the search strategy “Origin of SARS-CoV-2” on July 19, 2021, but fewer than 100 articles disclosed scientific evidence about the origins of SARS-CoV-2. As of September 27, 2021, there are nearly eight million scholarly articles in the CORD-19 dataset [10]. In other words, it is very difficult and time-consuming to screen scholarly articles about the origins of SARS-CoV-2 from a large amount of literature.

However, to alleviate the workload of an oracle in active learning and to smoothly run active learning, this study aims to prepare a *seed* dataset of annotated publications in advance. Fortunately, the “WHO-convened Global Study of Origins of SARS-CoV-2: China Part” [56] and several review articles about the origins of SARS-CoV-2, such as [55–60], provide valuable clues. The general idea is to determine a small collection of seed articles in the first place and then expand it on the basis of forward and backward citations of these seed articles.

More specifically, once [55–60] are chosen, the following steps are conducted on each article in this dataset to determine our seed articles. (a) The forward and backward citations are retrieved from the Dimensions database [61] with the Dimensions API according to the resulting DOI (digital object identifier) [62]. (b) The metadata information of each citation (such as title, abstract, publication time, publication venue, and so on) is fetched from the PubMed database with EFetch API after mapping DOI to PMCID or PMID. (c) The noisy citations are removed with three manually curated rules: the publication year must be later than December 2019, the research topic should be related to COVID-19, and the resulting article must have been peer-reviewed.

**Fig 2:**
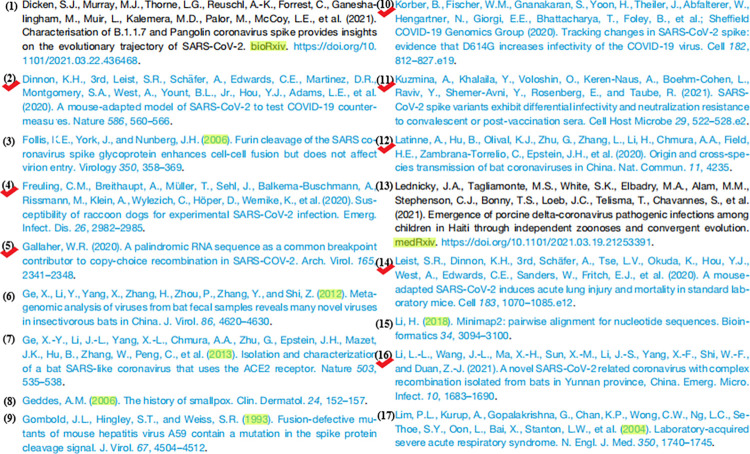
An illustrative example with a partial list of backward citations in [57].

To intuitively illustrate the rationale of our idea of collecting seed articles, we take a partial list of backward citations (references) in [57], shown in Fig 2, as an illustrative example. During the collection procedure, the following three rules are at work. First, since COVID-19 pneumonia broke out in December 2019, the articles published before December 2019 should not be related to the origins of SARS-CoV-2. Therefore, articles marked in yellow in Fig 2 are filtered out, such as (3), (6), (7), (8), (9), (15), and (17). Second, whether a scientific publication serves as a relevant instance or a nonrelevant instance, it should discuss COVID-19-related themes. Hence, one can rule out (13) in Fig 2. Last but not least, to focus on science, this study only considers peer-reviewed articles. In this way, preprints are excluded from further analysis, such as (1) in Fig 2. It is worth mentioning that we keep an eye on the status of each preprint by preprint-publication links [63]. Once it is published in a peer-reviewed venue, we will include it in our dataset. For the example in Fig 2, our seed dataset consists of (2), (4), (5), (10), (11), (12), (14), and (16).

Ultimately, this work collected 470 articles in total from 282 journals, covering the PubMed, Elsevier, and WHO databases. The involved fields include title, abstract, journal/conference, publication time and DOI. Table 1 shows the top 10 journals in our seed dataset, where Journal of Medical Virology ranks first in terms of the number of articles, followed by Nature, Science and Cell.

**Table 1:** The number of articles in the top 10 journals.

Journal	# of articles	Journal	# of articles
Journal of Medical Virology	18	Nature Communications	9
Nature	14	Lancet	7
Science	14	Infection, Genetics and Evolution	6
Cell	10	National Science Review	6
Emerging Microbes & Infections	9	Scientific Reports	6

#### Data annotation

Once our seed articles are determined, we need to attach a *relevant* or *nonrelevant* label to each article for active learning. Two annotators majoring in biology independently annotated all publications by reading the abstract and main body of every article. These two annotators were from the College of Biological Sciences and Biotechnology, Beijing Forestry University. Their research interests include the transmission and prevention of coronavirus. Furthermore, they have recently annotated the entities mentioned in the articles on COVID-19. Hence, they should be competent for the annotation work of our experiment.

To accurately annotate the articles in the seed dataset, we design an annotation guideline. It mainly gives several suggestions on which articles should be labeled as *relevant* or *nonrelevant*. The whole annotation process is mainly divided into two stages. In the first stage, to unify their understanding of the guideline, 50 of the same articles are assigned to these two annotators. The interannotator agreement is calculated with the multi-*κ* indicator [64]. The agreement between the two annotators was 80.2%. On closer examination, we find that the annotators have a different understanding of the articles mentioning intermediate hosts of SARS-CoV-2 (such as ferrets, cats, and dogs). Through extensive discussions, we argue that such articles should be relevant to the origins of SARS-CoV-2. Thereupon, the guideline is correspondingly revised. Then, according to the updated guidelines, they independently annotated the remaining articles in the second round as the final annotation results.

**Fig 3:**
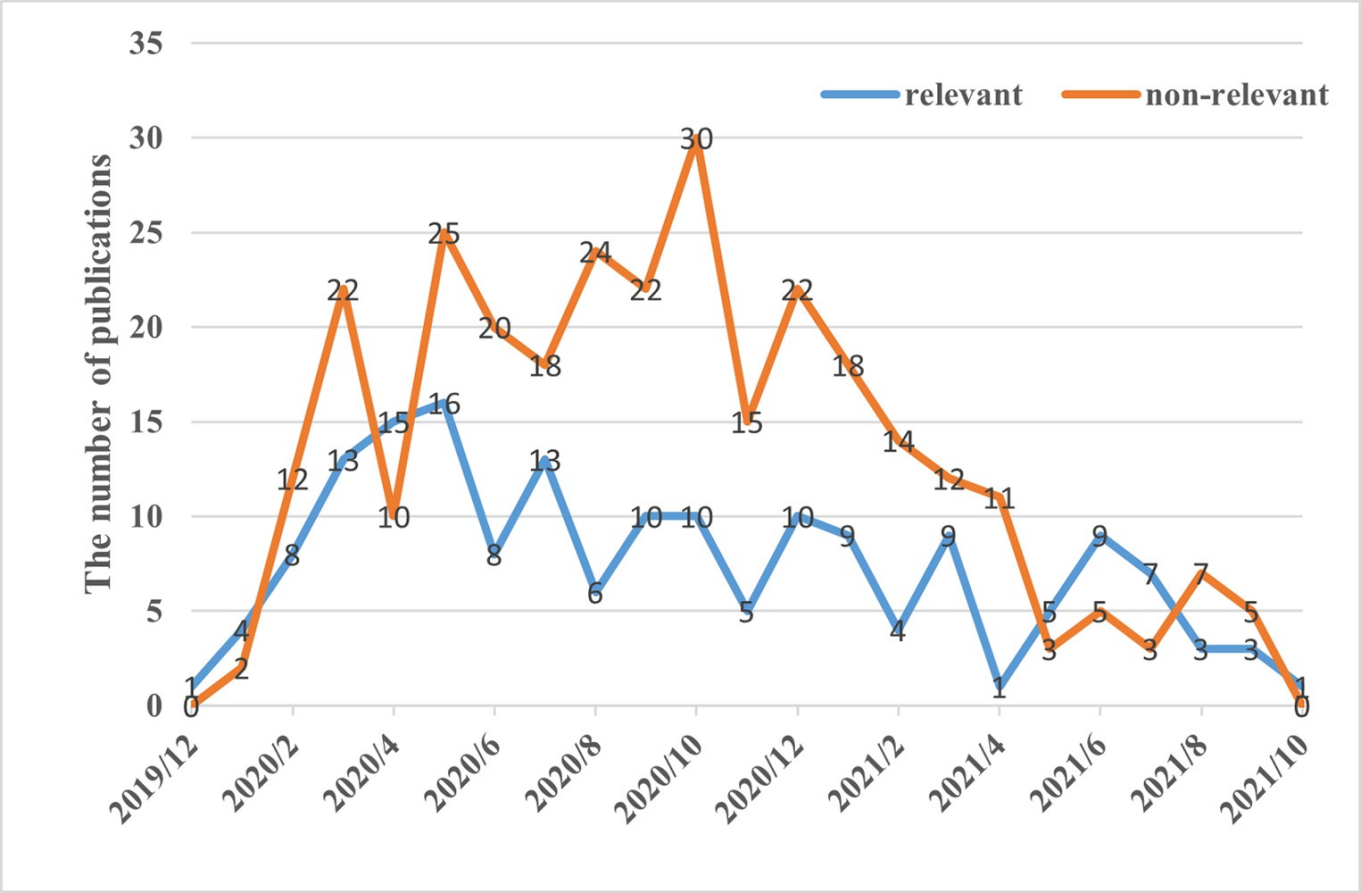
The trends of the number of relevant and nonrelevant publications with publishing time in our seed dataset.

Ultimately, our annotated corpus comprises 170 relevant articles (positive instances) and 300 nonrelevant articles (negative instances). Their trends with publishing time are shown in Fig 3. The publication time ranged from December 2019 to October 2021. Most articles were published between May 2020 and October 2020. The number of relevant and nonrelevant articles peaked in May 2020 and October 2020, respectively. These trends in Fig 3 are basically in line with the global trends in COVID-19 research [65].

### 3.2. Document representation

Another critical ingredient for screening scholarly articles about the origins of SARS-CoV-2 is how to represent a document with a fixed-length vector for active learning. Although many document representation methods are put forward in the literature, such as extensions of words to documents [66], convolution-based methods [67], and variational autoencoders [68], they are not able to leverage citation information between scientific documents. This greatly limits their representation power at the document level. Cohan et al. [69] developed a novel document representation approach, namely, SPECTER (Scientific Paper Embedding using Citation-informed TransforERs), through pretraining a transfer language model on the citation network of scientific documents. Thus, no task-specific fine-tuning is needed for our task, so this work prefers the SPECTER method.

It is worth noting that document embeddings with the SPECTER method on the basis of titles, abstracts and citation network are also released with each CORD-19 update [10]. More specifically, each scientific publication is represented with a 768-dimensional dense vector. To obtain these representations, we map each document in the seed dataset to that in the CORD-19 dataset through the resulting DOI [62].

## 4. Methods

**Fig 4:**
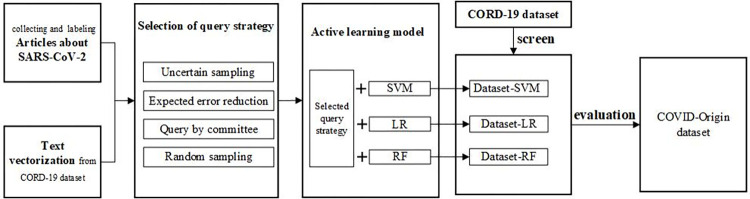
Research framework for screening articles about the origins of SARS-CoV-2.

To screen scholarly articles about the origins of SARS-CoV-2, our research framework, as shown in Fig 4, mainly consists of four modules. After collecting and labeling seed articles (cf. Subsection 3.1), we retrieve document representations of these articles from the CORD-19 dataset (cf. Subsection 3.2). Then, an active learning-based approach with SVM, LR or RF as a base classifier is deployed after optimizing the query strategy. On the basis of three tuned models with an active learning strategy, scholarly articles about the origins of SARS-CoV-2 are screened from the CORD-19 dataset and checked manually one by one for the top 1000 documents from each base classifier. In the end, a dataset about the origins of SARS-CoV-2, named the COVID-Origin dataset, is constructed. In the following subsections, the last three modules are described at length.

### 4.1. Query strategy

#### 1) Uncertain sampling

This query strategy selects the most uncertain instance for labeling. The most uncertain instance is referred to as the instance that the current classifier is most likely to make a mistake. Intuitively, such an instance can improve the performance of the model more efficiently. The uncertainty of an instance can be measured by information entropy. The more uncertainty an instance has, the greater its information entropy [70] is. Formally, this strategy can be expressed as follows:

$$x^{*}={argmax}_{x}-[Pr(relevant|x)\;\log\;Pr(relevant|x)+Pr(non\_relevant|x)log\;Pr(non\_relevant|x)]$$

Here, Pr(relevant|*x*) and Pr(non_relevant|*x*) represent the probability of *x* being classified into relevant and nonrelevant categories, respectively. When these probabilities approach 0.5, the resulting instance will be more likely to be selected.

#### 2) Expected error reduction

This strategy first estimates the generalization error of the current classifier and then sequentially evaluates the generalization error change that may be brought to the classifier if a new instance is added to the training set. Finally, it selects the instance for labeling that can reduce the expected generalization error the most. It is the generalization error minimization that enables this strategy to become an effective query strategy [71]. Nevertheless, this strategy brings a huge time cost due to its error reduction estimation, so it is inefficient for active learning on a large-scale dataset. Therefore, several approximate alternatives are proposed in the literature [71,72]. To speed up the process, the approximated error reduction in [72] is utilized in this study.

#### 3) Query by committee

In this strategy, multiple classifiers, namely, a committee of classifiers, are involved. The instance on which these classifiers disagree the most by voting will be chosen for labeling. The evaluation criteria for committee voting include entropy, Kullback‒Leibler divergence, and Jensen‒Shannon divergence. For simplicity, this study adopts voting entropy, which is defined formally as follows:

$$x^{*}={argmax}_{x}-\left[\frac{V(x,relevant)}{M}\log\frac{V(x,relevant)}{M}+\frac{V(x,non\_relevant)}{M}log\frac{V(x,non\_relevant)}{M}\right]$$

Here, *V*(*x*, relevant) and *V*(*x*, non_relevant) are the number of votes of the committee classifying instance *x* into relevant and nonrelevant categories, respectively. *M* is the total number of classifiers in a committee. When the votes of relevant and nonrelevant categories are approximately evenly distributed, the resulting instance will be more likely to be chosen.

### 4.2. Candidates in the CORD-19 dataset

Once our active learning-based approach with a proper query strategy is developed, it will be utilized to screen scientific publications from the CORD-19 dataset for follow-up real-world applications. In fact, the CORD-19 dataset covers scholarly articles on MERS-CoV, SAR-CoV and SARS-CoV-2. Therefore, before screening scholarly articles on the origins of SARS-CoV-2, articles that are not related to COVID-19 should be eliminated in advance.

In more detail, the following two steps are conducted. (1) This work extracts articles containing “COVID-19”, “2019-nCoV”, “SARS-CoV-2” or “coronavirus 2019” in the title or abstract. (2) The seed publications in Subsection 3.1 are excluded from the subset from the previous step. After these two steps, the candidates on the origins of SARS-CoV-2 are obtained for further screening with our active learning-based approach. For convenience, this subset is denoted as the *CORD-19 subset*.

### 4.3. Screening procedure

For ease of understanding, the pseudocode of our methodology is summarized in Algorithm 1. Our input includes the initial labeled set *S*, unlabeled set *S*_1_, query strategy, base classifier, and CORD-19 subset. Our algorithm mainly consists of the following three parts. (1) The classifier *f* is initialized with the labeled training set *S*_2_ in the first place (Line 3–4). (2) After an instance *I*_*ulab*_ chosen by the query strategy is annotated by an oracle, deleted from unlabeled set *S*_1_ and added to the labeled training set *S*_2_, the classifier *f* is retrained on the updated *S*_2_. This procedure is repeated until *f* reaches the best performance (Lines 5–10). (3) Finally, the tuned classifier *f* is utilized to screen the articles on the origins of SARS-CoV-2 from the CORD-19 subset (Lines 11–12).

**Algorithm 1.** Algorithms for screening articles about the origins of SARS-CoV-2.

1: **Input:** initial labeled set *S*, unlabeled set *S*_1_, query strategy, classifier *f*, and CORD-19 subset

2: **Process:**

3: Initialize the labeled training set *S*_2_ = *S*

4: Train an initial classifier *f* with the labeled training set *S*_2_

5: **Repeat**

6: Generate one new instance *I*_*ulab*_ from the unlabeled set *S*_1_ according to the query strategy

7: Get the label of *I*_*ulab*_ from an oracle

8: Update the labeled training set *S*_2_ and unlabeled set *S*_1_

9: Update the classifier *f* with labeled training set *S*_2_

10: **Until** the best performance of the classifier *f* is reached

11: Screen the publications on the origins of SARS-CoV-2 from the CORD-19 subset

12: **Output:** articles about the origins of SARS-CoV-2

## 5. Experimental results and discussions

The Python toolkit ALiPy [73] implements more than 20 commonly used active learning methods. Hence, it is utilized to screen articles on the origins of SARS-CoV-2. It is noteworthy that since a seed dataset of annotated publications is prepared ahead (cf. Subsection 3.1), our experiments are performed by simulating the labeling process by an oracle. That is, the resulting label of the document chosen by a query strategy is assumed to be unknown beforehand and must be assigned by an oracle during the active learning procedure.

### 5.1. Query strategy optimization

To tune the query strategy, our seed dataset is split randomly into a training set and a test set with a ratio of 7:3 and a similar relevant/nonrelevant distribution. That is, our training and test sets are made up of 329 and 141 instances, respectively. As shown in Algorithm 1, the base classifier *f* needs to be initialized. For this purpose, 8 instances are selected randomly from the training set as the initial labeled set *S*. Thus, our unlabeled set *S*_1_ is composed of the remaining 321 instances in the training set. In addition, apart from three query strategies (cf. Subsection 4.1), a random sampling query strategy is also used in this study. In fact, this query strategy is equivalent to the traditional supervised classification approach.

**Fig 5:**
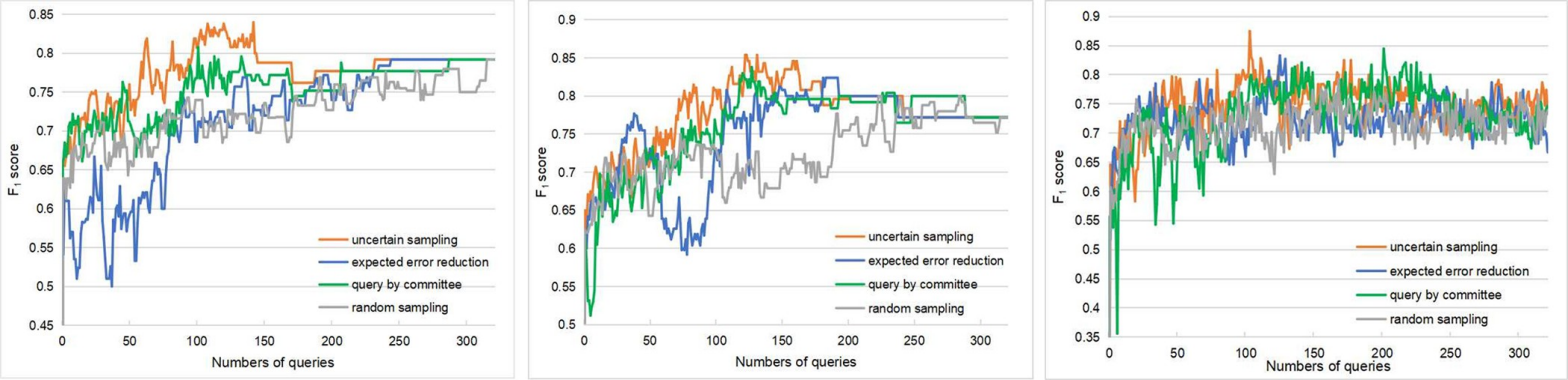
The performance of our active learning approach with support vector machine (a), logistic regression (b), and random forest (c) as the respective base classifiers in terms of the F_1_ score.

This study considers three base classifiers: support vector machines (SVM), logistic regression (LR), and random forest (RF). Fig 5 illustrates the performance of our active learning approach with different base classifiers and different query strategies on the test set in terms of the F_1_ score. From Fig 5, it is obvious that the active learning approach converges faster than the traditional supervised classification counterpart (viz. active learning approach armed with a random sampling query strategy). Among the three commonly used query strategies, the active learning approach armed with an uncertain sampling query strategy has the best performance, followed by the active learning approach armed with a query by committee query strategy. For base classifiers, the active learning approach with random forest (RF) as a base classifier has stable performance, regardless of the query strategy used.

To select an appropriate query strategy, this work simultaneously considers the F_1_ score and the number of queries when the active learning approach performs best on the test data, as reported in Table 2. Note that the number of queries is utilized to measure the workload saved by the active learning approach. A lower value indicates more workload saved. From Table 2, it is apparent that the performance of the active learning approach armed with an uncertain sampling query strategy is better than that of the active learning approach armed with other query strategies in terms of the F_1_ score and the number of queries. For example, after 103 queries, the combination of an uncertain sampling query strategy with random forest (RF) achieves the best F_1_ score. That is, only 103 labeled articles (except for 8 articles for initializing a base classifier) are needed to reach the maximum F_1_ score instead of all annotated articles. This indicates that approximately two-thirds of the workload for annotating articles can be saved. Hence, the active learning approach armed with an uncertain sampling query strategy is further used for screening scholarly articles about the origins of SARS-CoV-2 from the CORD-19 dataset in the next subsection.

**Table 2:** F1 score and numbers of queries when each model reached the maximum F1 score.

Base classifier	Query strategy	F_1_ score	# of annotated articles	
			Relevant	Nonrelevant
SVM	uncertain sampling	**0.840**	65	77
	expected error reduction	0.792	112	132
	query by committee	0.808	**39**	**62**
	random sampling	0.792	110	205
LR	uncertain sampling	**0.854**	**43**	**79**
	expected error reduction	0.824	67	113
	query by committee	0.838	49	77
	random sampling	0.799	81	142
RF	uncertain sampling	**0.875**	**47**	**56**
	expected error reduction	0.833	59	66
	query by committee	0.845	90	111
	random sampling	0.791	65	102

### 5.2. Screening articles about the origins of SARS-CoV-2

To screen articles about the origins of SARS-CoV-2, a comprehensive literature dataset, CORD-19 (2021-9-27 version), is utilized here. According to the criteria in Subsection 4.2, we can obtain a CORD-19 subset, which consists of 371,664 candidates in total. Then, our active learning approach with SVM, LR or RF as a base classifier independently assigns a posterior probability of the relevant category to each candidate. On the basis of posterior probabilities, the top 1,000 articles of each base classifier were checked manually one by one. This procedure is very similar to the annotation process in Subsection 3.1. Note that the articles in the CORD-19 dataset [10] come from multiple sources, such as WHO’s COVID-19 database, PubMed Central, MedLine, Elsevier and so on. To deduplicate publications, a conservative clustering policy in which any identifier (such as *doi*, *pmc_id*, *pubmed_id*, *arxiv_id*, *who_covidence_id*, and *mag_id*) conflict prohibits clustering was utilized. This enables many duplicative articles to appear in the CORD-19 dataset. This study further clusters these articles if any identifier matches and manually checks top articles in terms of posterior probabilities. Here, the top 1,000 articles actually correspond to the top ~1,800 articles in the original dataset.

**Fig 6:**
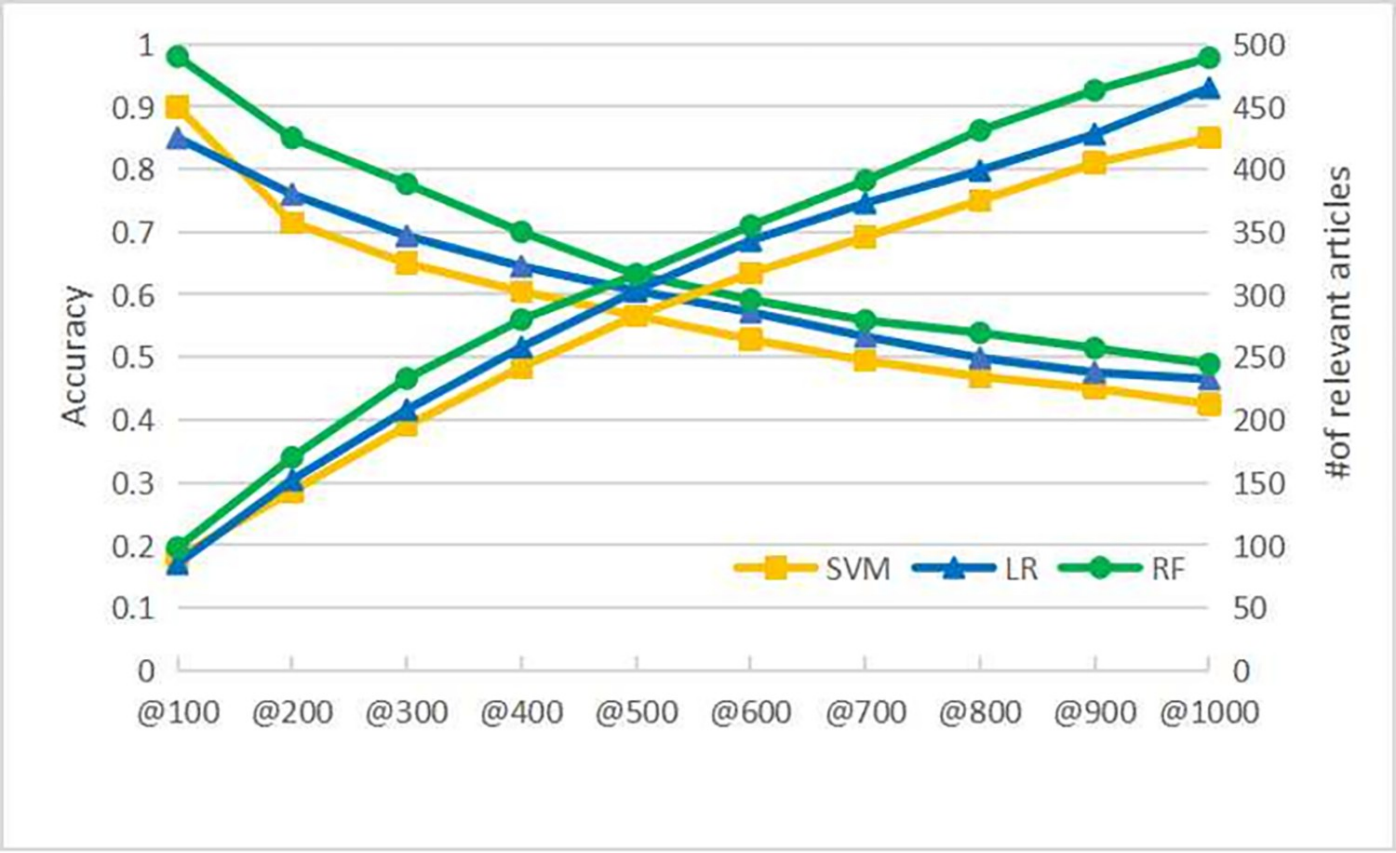
The performance of the top screened articles about the origins of SARS-CoV-2 from the CORD-19 dataset.

In this way, we can evaluate the performance of our approach in terms of accuracy, as depicted in Fig 6. The left vertical axis denotes the accuracy, and the right vertical axis is the number of relevant articles. Among the top 1,000 articles, the SVM, LR and RF base classifiers correctly screened 425, 465, and 489 articles, respectively. As the posterior probability of the relevant category decreases, the accuracy of the screened articles shows a downward trend. This is in line with our intuition. We take the top 200 articles as an example. The accuracies of all three base classifiers reach more than 70%, and the accuracy of the RF classifier even exceeds 80%. This indicates that our active learning-based approach for screening articles about the origins of SARS-CoV-2 is feasible.

**Fig 7:**
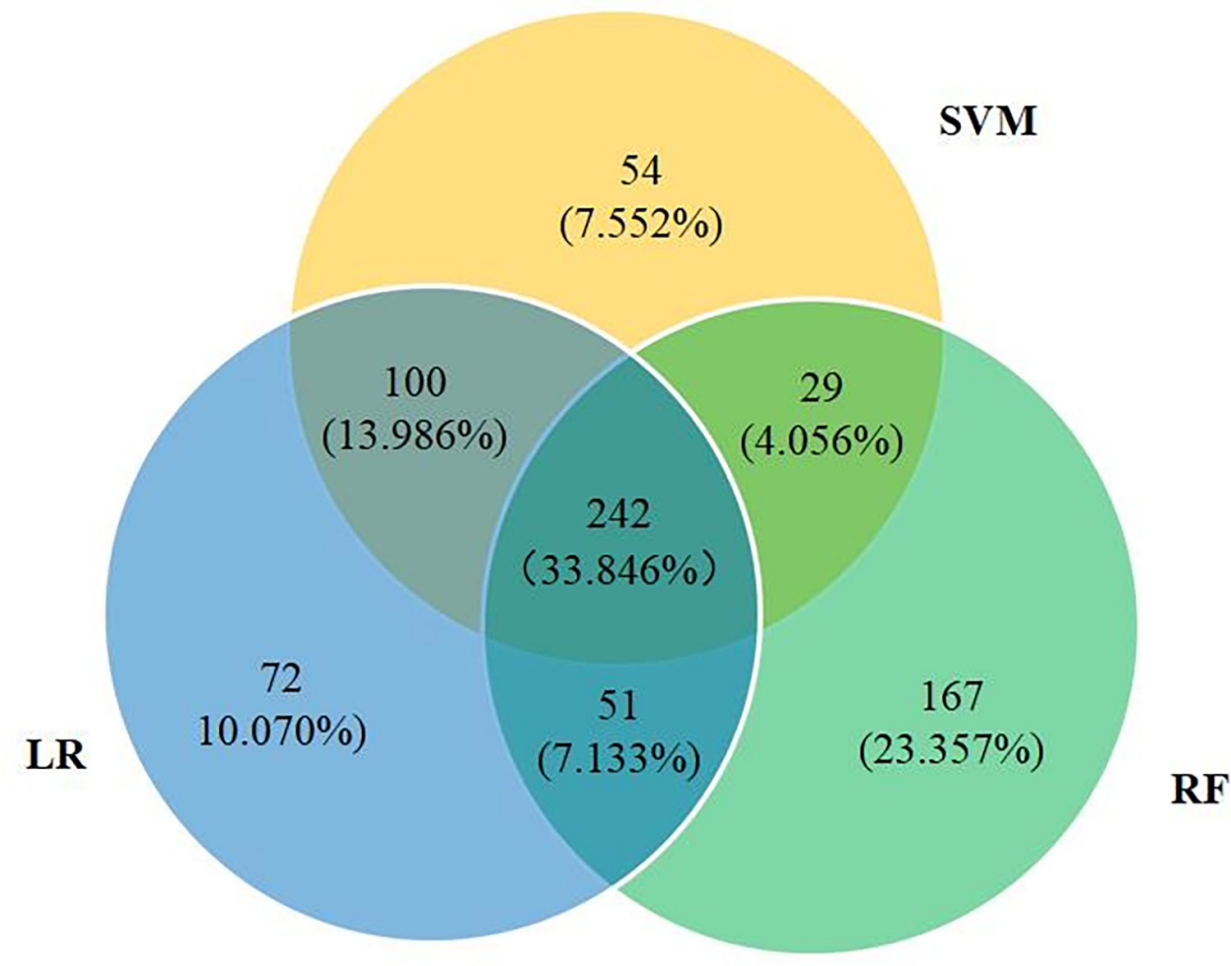
The overlapping shares of relevant articles screened by three base classifiers.

In total, 715 unique articles were screened among the top 1,000 scholarly articles by three base classifiers. That is, the articles screened by three base classifiers overlap greatly. Fig 7 depicts the overlapping shares of relevant articles screened by three base classifiers. It is not difficult to see that the articles screened simultaneously by three base classifiers account for 33.846%, and those screened by two classifiers account for at least 59.021%. This indicates that each base classifier has its pros and cons and cannot serve as an alternative to the others. In real-world applications, it is better to screen scientific publications on the origins of SARS-CoV-2 with multiple base classifiers in our framework (cf. Fig 4).

## 6. Conclusions

The outbreak of COVID-19 has disrupted people’s daily lives and work for nearly two years. To completely solve this epidemic, one of the most important tasks is to trace the origins of SARS-CoV-2. Due to the complexity of traceability work, the origins of SARS-CoV-2 are still inconclusive. It is necessary for researchers to build a full picture of previous studies on the origins of SARS-CoV-2 in advance and then to conduct further investigations. However, there is currently no comprehensive literature dataset on the origins of SARS-CoV-2 that can be used by researchers. Therefore, to bridge this gap, this study is devoted to screening scholarly articles about the origins of SARS-CoV-2 from large numbers of scientific publications.

For this purpose, we propose an active learning-based approach that can quickly screen articles with better accuracy and save the labeling workload of human annotators. In more detail, after collecting and labeling a small seed dataset of articles, we develop the active learning-based approach with three query strategies and three base classifiers (SVM, RF, and LR). Extensive experiments show that our approach has better performance than its traditional counterparts, and the uncertain sampling query strategy performs best among the three strategies. To quantitatively evaluate the performance of the three base classifiers, we manually checked the top 1,000 articles one by one in terms of posterior probabilities. In the end, three classifiers screened 425, 465 and 489 relevant articles. In total, there were 715 unique articles, more than 50% of which were screened by at least two base classifiers.

However, there is still room to improve our approach. For example, only three query strategies are taken into consideration in this work. In the near future, other query strategies, such as the graph density query strategy [74], will be used to screen scholarly articles on the origins of SARS-CoV-2. In addition, due to the pros and cons of each base classifier, ensemble learning will be utilized in our next work as a base classifier for our active learning-based approach.
